# Genomics and functional genomics in *Leishmania* and *Trypanosoma cruzi*: statuses, challenges and perspectives

**DOI:** 10.1590/0074-02760200634

**Published:** 2021-03-29

**Authors:** Daniella C Bartholomeu, Santuza Maria Ribeiro Teixeira, Angela Kaysel Cruz

**Affiliations:** 1 Universidade Federal de Minas Gerais, Departamento de Parasitologia, Belo Horizonte, MG, Brasil; 2 Universidade Federal de Minas Gerais, Departamento de Bioquímica e Imunologia, Belo Horizonte, MG, Brasil; 3 Universidade de São Paulo, Faculdade de Medicina de Ribeirão Preto, Departamento de Biologia Celular e Molecular, Ribeirão Preto, SP, Brasil

**Keywords:** Leishmania, Trypanosoma cruzi, genomics, transcriptomics, functional genomics

## Abstract

The availability of Trypanosomatid genomic data in public databases has opened myriad experimental possibilities that have contributed to a more comprehensive understanding of the biology of these parasites and their interactions with hosts. In this review, after brief remarks on the history of the *Trypanosoma cruzi* and *Leishmania* genome initiatives, we present an overview of the relevant contributions of genomics, transcriptomics and functional genomics, discussing the primary obstacles, challenges, relevant achievements and future perspectives of these technologies.

The Trypanosomatidae family comprises obligate protozoan parasites from the Kinetoplastida order, including human and livestock disease agents. *Trypanosoma cruzi*, *Trypanosoma brucei* and *Leishmania* species are three parasites representative of this family and are the etiological agents of Chagas disease, African trypanosomiasis and leishmaniasis, respectively. Trypanosomatid genomics was born at FIOCRUZ in 1994. The Parasite Genome Network Planning Meeting was held in April in Rio de Janeiro, and it was sponsored by FIOCRUZ and the World Bank/WHO (World Health Organization) Special Program for Research and Training in Tropical Diseases (TDR). The genome projects of *T. cruzi*, *T. brucei*, and *Leishmania major* were launched at this meeting. The Trypanosomatids Genome Initiative gathered several laboratories from different countries to sequence reference strains of *T. brucei*, *T. cruzi*, and *L. major*. Eleven years were needed to reach sequencing coverage and annotation status before the Tritryp genomes were considered complete.^1^ ^,^ ^2^ ^,^ ^3^ ^,^ ^4^ Financial constraints, sequencing limitations of Sanger technology, the computational capacity of tools available at the time, and the limited number of groups with high-throughput sequencing capacity and computational expertise explain the length of time needed to complete the task.

In this review, we will focus on *T. cruzi* and *Leishmania* omics, advances and challenges. The major achievements in these areas are depicted in Figure. However, a brief word on the first publications and the combined efforts on the Tritryps is warranted. *L. major* and *T. brucei* genomes were sequenced and assembled using a combination of a clone-by-clone approach and individual chromosome shotgun sequencing,^3^ ^,^ ^4^ in which the genome assembly is local (clones of tens of kilobases or chromosomes) and therefore not so complex, resulting in a high-quality assembled genome. Conversely, for *T. cruzi*, the clone-by-clone approach was impractical due to the high repetitive content of the genome, and obtaining chromosome-specific libraries was highly difficult to achieve due to the highly complex parasite karyotype. Therefore, a whole genome shotgun strategy was chosen to sequence the genome of *T. cruzi*.^2^ In this strategy, the entire genome is fragmented, and no positional information of the individual sequences is available to the assembler algorithms to reconstruct the genome sequence. Compared to the *T. brucei* and *L. major* genomes, for *T. cruzi*, the assembly step was notably more complicated. Additionally, CL Brener, the reference strain for the *T. cruzi* genome project, is a hybrid between two divergent lineages (TcII and TcIII). This added another layer of complexity to the genome assembly because of the similarity between the two parental haplotypes. The initial assembly attempt resulted in many pseudogenes, since the assembler merged sequences from the two parental haplotypes. More stringent assembly parameters were then applied in an attempt to assemble the two haplotypes separately, but as a drawback, this strategy resulted in a more fragmented genome assembly. In contrast to *L. major* and *T. brucei*, no closure step to resolve gaps was applied in the CL Brener *T. cruzi* genomic assembly. All these aspects contribute to the difference in the quality of the first *T. cruzi*, *T. brucei* and *Leishmania* reference genomes available in public databases.

**Figure:**
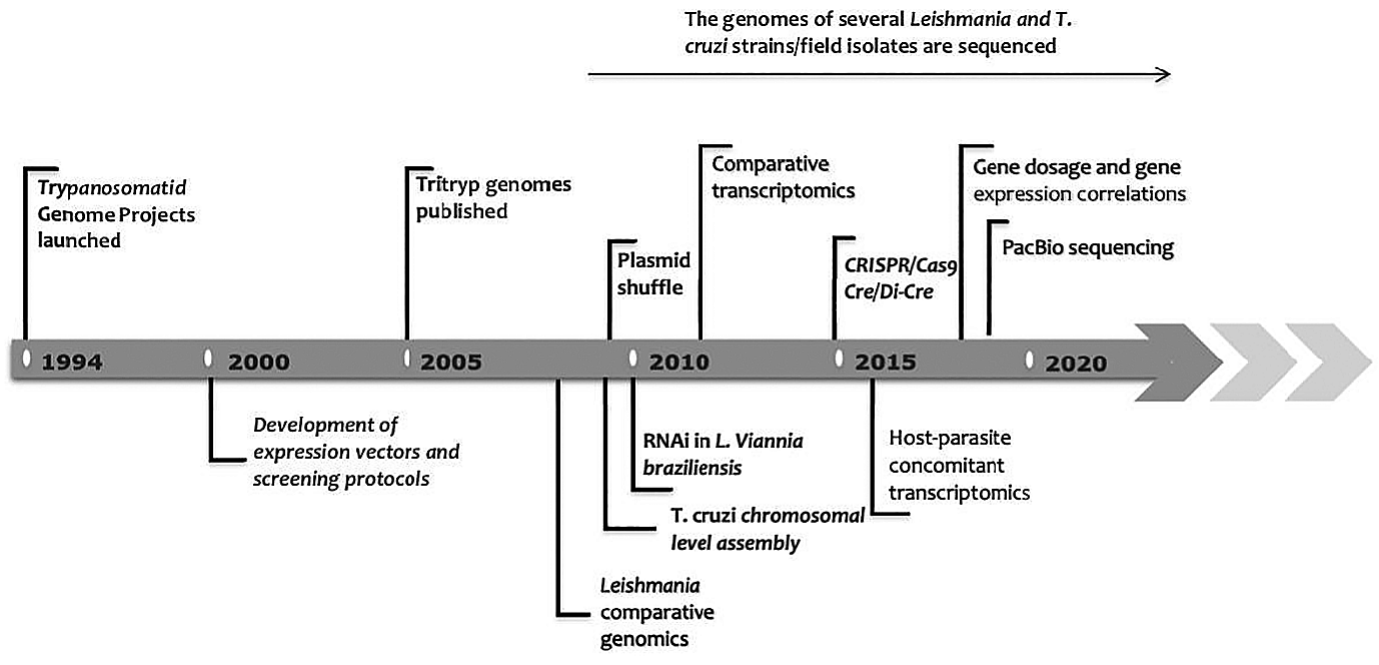
Timeline showing some of the major achievements in the areas of genomics, transcriptomics and functional genomics in *Leishmania* and *Trypanosoma cruzi*. Important genomic initiatives launched before 1994 were included in a comprehensive and recent review by Ramírez, 2020.^5^

Although structural three-way genome comparisons among the Tritryps were somewhat limited due to the *T. cruzi* genome fragmentation, gene content comparisons among the three parasites revealed a core genome of ~6,200 genes distributed in syntenic polycistronic units containing a large number of genes transcribed from the same DNA strand. Despite evolutionary distance, the extent of syntenic blocks comparing *T. brucei* and *Leishmania* genomes is impressive; 68 and 75% of the annotated genes are retained in the same genomic context, and almost all of these genes, 94%, are part of the core proteome of the three trypanosomatids.^1^ In terms of gene content, the total number of annotated protein-coding genes was similar for *T. brucei* and *Leishmania*, ~8,000, and significantly higher in *T. cruzi*, 12,000. Notably, only 32, 26 and 12% of species-specific genes were identified in *T. brucei*, *T. cruzi* and *Leishmania*, respectively; their predicted functions are clearly connected to these organisms’ most clear biological differences.^1^

Shortly after the publications of the Tritryp genomes, the first comparative analysis of *Leishmania* genomes was conducted between *L. (Leishmania) infantum* and *L. (Viannia) braziliensis* with the available *L. major* genome in 2007.^6^ These species are associated with different disease outcomes; typically, *L. major* leads to uncomplicated cutaneous disease (LCL), *L. infantum* leads to visceral disease (VL) and *L. braziliensis* is responsible for the morbid mucocutaneous forms (MCL and LCL).^6^ As expected, the study demonstrated the genomes to be highly conserved at the content and synteny levels.^6^ This comparative analysis contributed to unraveling similarities and differences due to the restricted number of species-specific genes detected; the highest number of *L. braziliensis*-specific genes was 49.^6^ Notably, this study revealed that in contrast to the two *Leishmania* from the subgenus *Leishmania*, *L. braziliensis* from the *Viannia* subgenus contains an intact RNA-mediated interference pathway. The RNAi pathway was subsequently shown to be functional and rapidly applied to gene function studies.^7^ ^,^ ^8^

In 2009, a critical contribution to the CL Brener *T. cruzi* genome sequence was the generation of a chromosomal-level assembly by Weatherly et al.^9^ A total of 41 pseudochromosomes for each CL Brener haplotype, TcII and TcIII, were assembled based on the scaffolds generated in 2005, BAC end sequences and synteny maps with the *T. brucei* genome.

*Advances and difficulties in data generation and analysis* - For the first descriptions of the *T. brucei*, *Leishmania* and *T. cruzi* genomes generated in 2005 and 2007, Sanger sequencing technology was used. Since then, we have seen a notable improvement in new sequencing technologies, which provide considerably higher throughput and accurate short-read sequencing, as well as noisy long-read sequencing. The quality of long reads can be improved by sequencing a single template multiple times using hairpin adaptors or using high-quality short Illumina reads to correct long PacBio or Nanopore reads. The use of high-quality consensus long reads (10-100 kb) dramatically improves genome assembly, allowing the resolution of many repetitive regions, phasing alleles, distinguishing highly homologous sequences, and identifying structural variants, such as deletions, duplications, insertions, inversions, and translocations. Recently, Trypanosomatid genome projects have used both technologies, with long-read sequencing offering assembly continuity and short-read sequencing providing higher sequence accuracy. The genomes of several *T. cruzi* strains, such as Berenice, Dm28c, Bug2148, and TCC, were assembled using long reads or a combination of long and Illumina reads, enabling a reduction in the number of sequences generated and improving the contiguity of the assembled genome.^10^ ^,^ ^11^ ^,^ ^12^ More recently, high-quality chromosomal level assemblies of Y and Brazil strains were obtained using a combination of long and Illumina reads and Hi-C and Chicago proximity-ligation libraries, improving genome contiguity and assembly accuracy.^13^ The improvement of *T. cruzi* genome assemblies confirmed the compartmentalisation of the parasite genome’s organisation. Berná et al. proposed the occurrence of two compartments named “core” and “disruptive” that differ in GC content and gene composition.^10^ The core compartment comprises conserved and hypothetical genes and is syntenic with the *T. brucei* and *Leishmania* genomes, while the disruptive core is composed of multigene families encoding MASP, TcMUC, and trans-sialidase variable surface proteins. Other multicopy gene families, such as DGF-1, GP63, and RHS, are evenly distributed throughout the genome.^10^ Wang et al.^13^ proposed the occurrence of three genomic compartments: (i) one corresponding to the disruptive compartment described by Berná encompassing rapidly evolving gene families associated with immune evasion; (ii) a second compartment containing gene families, such as TcSMUG, expressed in the vector stage, distributed as tandem arrays and displaying minimal diversification; and (iii) a third compartment containing all other core genes. Regardless of the number of compartments, it is clear that some *T. cruzi* genomic regions evolve under strong selective pressure for diversification, while others exhibit low diversity.^10^ ^,^ ^13^ These improvements in *T. cruzi* genome assembly have allowed a better understanding of the parasite genomic organisation and disclosed the complete repertoire of genes encoding surface proteins involved in several critical host-parasite interactions.

It is very important to keep in mind, however, that despite the many advances, the current sequencing and genome assembly approaches do not have enough resolution to generate the complete genome sequencing of diploid organisms. Only more recently have assembler algorithms devoted to dealing with haplotype reconstruction, i.e., set of single nucleotide variations that distinguish chromosomal sequences from their homologous pairs, been developed.^14^ ^,^ ^15^ Although the use of PacBio and Nanopore long reads by these assemblers improves the reconstruction accuracy, haplotype assembly remains a complex computational task. Therefore, for most genomes, only a mosaic haploid representation of the genome sequence is available in public databases. This is especially relevant for *T. cruzi* and *Leishmania* genomes, as they are prone to extensive chromosome copy number variations.^16^ ^,^ ^17^

Although complete haplotype reconstruction is still a challenge, computational methodologies based on next-generation sequencing (NGS) data have been developed to estimate genome ploidy and chromosome “somies”, such as allelic frequency analyses and read depth coverage (RDC) (the average number of times a base of a genome is sequenced). Both analyses require chromosomal-level assembly of a reference genome and high RDC provided by NGS data.

RDC analyses are based on the assumption that coverage is directly related to copy number. Basically, the NGS reads are mapped on a reference chromosome sequence, and the number of reads covering each position or gene is computed. The number of copies of a given gene, genomic region, or chromosome can then be estimated by dividing the median RDC in that region by the median RDC of the entire genome. In the case of very repetitive genomes, such as *T. cruzi*, instead of using RDC of the whole genome as a normaliser, it is more reliable to use the RDC of unique (single copy) genomic regions because the reads that map to repetitive regions have low mapping confidence.^17^ It is also essential to control for RDC variations along each chromosome sequence to exclude segmental duplications/deletions that could bias the median RDC value. In summary, an increase or decrease in the median RDC of a chromosome compared to the whole genome (or single-copy regions) indicates the gain or loss of chromosomal copies, respectively. For a diploid genome, if the ratio chromosome median RDC/genome median RDC is close to 1, the chromosome is disomic; if this ratio is 1.5, or 2, the chromosome is trisomic or tetrasomic, respectively, and so on.

Allelic frequency analysis is another NGS-based approach to estimate ploidy. As in RDC analysis, the initial step consists of mapping NGS reads on a reference genome. Next, the frequency of heterozygous SNPs that have up to two and only two variants is computed. The chromosome somy can then be inferred by computing the proportion of reads that support each variant. In a disomic chromosome, the same number of reads are expected to support each heterozygous SNP, resulting in a peak allele frequency of 0.5 (50%). For trisomic chromosomes, the expected allele frequency is a combination of 0.33 and 0.66 frequencies, while in tetrasomic chromosomes, frequencies of 0.25, 0.5 and 0.75 are expected. For monosomic chromosomes, no SNP should be identified. Whenever possible, both RDC and allele frequency approaches should be used to estimate somy, so independent evidence can be evaluated. However, the low frequency of allele polymorphisms in many *Leishmania* species/isolates may preclude allele frequency analysis, and the chromosome somy based on NGS data is mainly estimated by the RDC approach.

These NGS-based methodologies and fluorescence *in situ* hybridisation (FISH) analyses have been recently used to infer ploidy in *T. cruzi* and *Leishmania*, revealing that aneuploidy seems to be the rule in these organisms.^16^ ^,^ ^17^ ^,^ ^18^ As many genomes of *T. cruzi* strains and *Leishmania* species/strains have been sequenced, it is now clear that these parasites display extreme chromosome copy number variations (CCNV), a phenomenon that affects many, if not all, chromosomes. Even within a given parasite population, FISH analysis of *Leishmania* revealed variable chromosome ploidy among individual cells generating intrastrain heterogeneity, which is a pattern known as mosaic aneuploidy.^18^ The question that emerged from these studies is what is the biological meaning of these findings?

Gene enrichment analysis of chromosomes consistently supernumerary in *T. cruzi* ^17^ suggested a possible correlation between chromosome copy number and dosage of genes involved in crucial parasite biological processes. As an example, chromosome 31, which was found to be supernumerary in the large majority of strains and isolates analysed, is enriched with genes involved in glycosylation and glycan biosynthesis, especially the enzyme UDP-GlcNAc-dependent glycosyl transferase, which is involved in mucin glycosylation and crucial for many parasite interactions with invertebrate and vertebrate hosts.^17^ ^,^ ^19^

Recent studies have disclosed direct evidence for the link between aneuploidy and gene expression in *Leishmania*. For instance, Dumetz et al.^20^ examined aneuploidy and gene expression in *L. donovani* strains under different *in vitro* and *in vivo* conditions. The results revealed aneuploidy alterations among the different culture and infection conditions and a positive correlation between chromosome copy number and corresponding transcript levels, although independent aneuploidy mechanisms of gene regulation were observed for chromosomes 5, 8 and 31. As the control of gene expression in *Leishmania* operates at the posttranscriptional level, changes in aneuploidy have emerged as a major adaptative strategy exploited by *Leishmania* to modulate expression by altering gene dosage, allowing rapid adaptation to changing environments.^21^

As more layers of novel information arise from comparison of *Leishmania* field strains and species from different geographical endemic areas and long-maintained laboratory strains or drug-resistant versus drug-sensitive strains, an increasing number of questions emerge. Aneuploidy, chromosome-variable somy, loci-restricted DNA amplification, and expansion and deletions in gene arrays contribute together and to a different extent to gene copy number variation. Some paradigmatic studies have revealed the complexity of the genomic adaptation of these parasites to the field environment.^22^ ^,^ ^23^ These studies and others indicate that the genomic plasticity of *Leishmania* plays a role in determining phenotypic variation and fitness gain in response to environmental changes and challenges.

Whole-genome sequencing has also provided new insights into the population genetic structure of Trypanosomatids, demonstrating that facultative sex is a common reproductive strategy in this group of parasites. Sequencing of 45 TcI *T. cruzi* field isolates from vectors and mammalian hosts in southern Ecuador revealed that sexual meiotic reproduction occurs in some groups, while in others, long-term clonality remains after episodic hybridisation events. These distinct groups seem to be genetically segregated even though they may cooccur in the same invertebrate and vertebrate hosts.^23^ The site of *T. cruzi* genetic exchange, whether it occurs in the vector or mammalian host, remains unclear. On the other hand, in *Leishmania* and in *T. brucei*, sexual reproduction has been previously demonstrated to occur in their vectors.^24^ ^,^ ^25^ The occurrence of recombination in Trypanosomatids implies that genetic traits associated with, for instance, virulence and drug resistance are not restricted to specific subdivisions but instead can be recombined and transferred, affecting parasite fitness and adaptation to new niches.^26^ ^,^ ^27^

*Genome and transcriptome information catalysing functional studies* - A primary goal of any genome research is the elucidation of gene function and the mechanisms involved in the control of gene expression. The combination of genomics and transcriptomics data and analyses is greatly increasing our understanding of molecular mechanisms of parasite adaptation to distinct host environments. Due to recent advances in genomics, we are progressing toward a more complete understanding of genome structure, gene function and, ultimately, how the information encoded in an organism’s DNA guides the synthesis of all RNA and protein molecules to determine the characteristics of a living cell. For Trypanosomatids, such studies also allow us to understand species differences that help us to make rational choices in functional studies of pathways and gene networks relevant to parasite survival.^20^ ^,^ ^28^ ^,^ ^29^

The ideal scenario for evaluating parasite gene expression within natural environments and/or concomitantly analysing profiles of gene expression of hosts and parasites is currently possible. Of note, these analyses depend on robust computational tools and genome data of parasites and hosts. Quite informative studies on parasite-host interactions have been reported that highlight the main differences between the transcriptomes of *Leishmania* sand fly stages^30^ ^,^ ^31^ by comparing organisms maintained *in vivo* and *in vitro*. A plethora of data on differential gene expression and gene ontology analysis permit investigation of the host and parasite responses during infection; the available studies include a comparative analysis of different species of *Leishmania* in *in vitro*-infected human macrophages and a challenging analysis of pauci-parasitic *L. braziliensis* human lesions. Similar studies simultaneously capturing the transcriptome dynamics in the parasites and host cells were reported for an infection time course of human fibroblasts with two different *T. cruzi* strains, showing contrasting virulence phenotypes.^32^ ^,^ ^33^ ^,^ ^34^ Such studies are invaluable for understanding parasite evasion, host response, and parasite-driven host cell subversion.

Whereas fast-evolving sequencing platforms and bioinformatic tools are quickly overcoming most of the challenges faced by researchers during genome sequencing, assembly and annotation, as well as transcriptome analyses, gene manipulation protocols are only now experiencing significant improvements that will provide more significant advances in functional genomic studies.

Genome manipulation tools and protocols are essential for any study aiming to determine gene function and identify the elements involved in gene expression. In the first report describing gene deletion in *Leishmania*, published in 1990, it was demonstrated that the single allele of DHFR-TS can be replaced with high efficiency by homologous recombination after transfection of *L. major* promastigotes with linear dsDNA containing sequences homologous to the target gene.^35^ In *T. cruzi*, studies based on gene manipulation began in 1991, when Lu and Buck transfected epimastigotes with a plasmid vector in which a sequence encoding the spliced leader (SL) repeat was inserted upstream from the bacterial chloramphenicol acetyltransferase (CAT) gene. Surprisingly, these authors succeeded in detecting CAT activity in lysates from transfected parasites even in the absence of correct information regarding the sequences required for gene expression in *T. cruzi*. Similar to *Leishmania*, the first gene deletion experiment in *T. cruzi* described the disruption of only one allele of the gene encoding the flagellar adhesion molecule GP72 by homologous recombination.^36^

The study of mechanisms involved in gene expression in *T. cruzi* and *Leishmania* yielded many surprises about the Trypanosomatid family (for a review, see^37^). Although studies on the variant surface glycoprotein (VSG) and tubulin genes showed identical 35 nucleotide sequences added on the 5’ ends of different mRNAs from *T. brucei* since 1985,^38^ the coupled trans-splicing and polyadenylation mechanisms were elucidated only after gene manipulation protocols became well established in this parasite.^39^ A remarkable series of experiments then revealed that this group of early branched eukaryotes has developed peculiar transcription and mRNA processing mechanisms. Unlike most eukaryotes that transcribe their genes into monocistronic pre-mRNAs containing exons (coding sequences) and introns (mostly noncoding sequences), almost all trypanosome protein-coding genes are intronless and transcribed into polycistronic pre-mRNAs, which require processing into mature mRNAs through “trans-splicing” and polyadenylation reactions. In addition to a poly-A tail at their 3’ end, every trypanosomatid mature mRNA possesses a common sequence at its 5’ end, which is capped and contains the same 39 nucleotides (not 35 nucleotides as postulated in 1985) named the spliced leader (SL). Once polycistronic mRNAs are transcribed, coupled trans-splicing and polyadenylation machinery, driven by polypyrimidine-rich region sequences present within intergenic regions (IRs), promotes the cleavage between the coding sequences and the simultaneous insertion of the poly-A tail and SL on upstream and downstream genes, respectively. Another bizarre discovery is the enzyme that transcribes trypanosome mRNAs. *T. brucei* possesses all three RNA polymerases and RNA polymerase II (RNA pol II) is responsible for the transcription of most protein-coding genes, as in any eukaryote; however, two groups of *T. brucei* protein-coding genes, VSG and PARP, are transcribed by RNA polymerase I (RNA pol I), which, in most eukaryotes, transcribes exclusively ribosomal RNA genes. Surprisingly, in contrast to RNA polymerase I promoters, no consensus sequences for RNA pol II promoters have been identified in trypanosomatids, with the exception of genes encoding SL transcripts. Therefore, to express a foreign gene in *T. cruzi* or in *Leishmania* spp., IR containing polypyrimidine-rich sequences must be inserted upstream and downstream from the exogenous gene in the expression vector to allow correct trans-splicing and polyadenylation. Moreover, as shown in early experiments, it is possible to express, albeit at low levels, a foreign gene in *T. cruzi* or *Leishmania* without a transcriptional promoter. However, even though transcription of endogenous protein-coding genes by RNA pol I has been described only in *T. brucei*, RNA pol I promoters derived from the ribosomal RNA locus have been successfully used to increase the expression levels of foreign genes in both *T. cruzi* and *Leishmania*.^40^

Since the early 1990s, major advances in our understanding of the mechanisms controlling gene expression in *T. cruzi* and *Leishmania* have been made due to experiments involving transfection of exogenous DNA into parasites by conventional electroporation. More recently, a new transfection methodology named nucleofection using the Amaxa Nucleofector system resulted in considerably improved transfection efficiency. These gene manipulation studies, performed mainly with *T. cruzi* epimastigotes and *Leishmania* promastigotes, also revealed unique gene expression mechanisms. As a consequence of polycistronic transcription of protein-coding genes, which are constitutively transcribed, as well as the absence of sequences controlling transcription initiation, it became clear that *T. cruzi* and *Leishmania* genes must be posttranscriptionally regulated in response to the changes in the environment these parasites face during their life cycles. Regulatory sequences that are present mainly in the 3’ UTRs of mRNAs act as protein-binding sites, and the regulatory sequences in the 3’ UTR and RNA binding proteins (RBPs) are key factors that modulate individual mRNA levels during the parasite life cycle.^37^ Even before the availability of the whole genome sequence, a combination of experimental approaches, including bioinformatics analyses and parasite transfection with reporter genes, were used to identify elements in the 3’ UTRs of several genes that control mRNA abundance, as well as translation. The identification of trans-acting elements that bind to mRNA regulatory motifs requires additional strategies, such as RNA affinity chromatography with parasite protein lysates. The development of knockout or knockdown mutants is essential to directly test the role of an RBP on global gene expression. In contrast to *T. brucei*, in which the discovery of the RNAi machinery allowed the testing of several RBPs, the lack of RNAi machinery in *T. cruzi* ^41^ and all species of *Leishmania* ^7^ from the subgenus *Leishmania* delayed the studies in *T. cruzi* and *Leishmania*.

Gene knockout experiments are powerful tools used to determine gene function. In contrast to *T. brucei*, protocols that allowed reverse genetic manipulation of the *T. cruzi* and *Leishmania* genomes have been used in a limited number of studies that have revealed the function of parasite genes and their roles in complex interactions with parasite hosts. New protocols describing alternative methodologies to facilitate genetic manipulation in *T. cruzi* are indeed desirable. A major improvement that has allowed for better control of genetic manipulation in these parasites was the development of inducible expression of gene products using the tetracycline repressor. This system, initially developed for *T. brucei*, requires the generation of transgenic parasites that express the tetracycline repressor from *Escherichia coli*. Although tetracycline-dependent expression of a reporter gene cloned downstream from the tet operator^42^ has been successfully described in *T. cruzi*,^41^ to date, this methodology has not been widely used to control exogenous gene expression in either *T. cruzi* or *Leishmania*. We now have a much broader collection of vectors that can be used to perform genetic manipulation in *T. cruzi* and *Leishmania*, including vectors containing sequences that allow the integration of the transfected gene into different loci of the parasite genome. The most recent improvement in our capacity to perform studies involving genome manipulation in these parasites came with CRISPR/Cas9 technology. Due to the efforts made by a somewhat limited number of research groups, highly efficient gene manipulation protocols based on CRISPR/Cas9 technology have been developed for *T. cruzi* and *Leishmania*, enabling the generation of mutant parasite cell lines with targeted sequence modifications in their genomes in days instead of months.^43^ ^,^ ^44^ ^,^ ^45^ ^,^ ^46^ ^,^ ^47^ ^,^ ^48^ Using CRISPR/Cas9, alterations in the genome can be created more precisely and more efficiently due to the induction of site-specific double-strand breaks (DSBs) caused by the RNA-guided Cas9 endonuclease. If a donor DNA template is provided, DSBs are efficiently repaired by the parasite homologous recombination DNA repair machinery, allowing not only target gene deletion but also sequence replacement that facilitates characterisation of functional domains in protein-coding genes as well as noncoding regulatory sequences.^49^ Importantly, together with high-throughput DNA/RNA sequencing technologies, CRISPR/Cas9 genome editing will allow genome-wide studies, which will produce a shift towards a global perspective on gene function studies with the examination of the expression and biological roles of gene networks in different stages of the parasite life cycle and under different contexts of host parasite interactions.
